# Causal Link between Ventricular Ectopy and Concussion

**DOI:** 10.1155/2020/7154120

**Published:** 2020-06-03

**Authors:** J. Patrick Neary, Jyotpal Singh, Jonathan P. Christiansen, Taylor A. Teckchandani, Kirsty L. Potter

**Affiliations:** ^1^Faculty of Kinesiology & Health Studies, University of Regina, 3737 Wascana Parkway, Regina, SK S4S 0A2, Canada; ^2^University of Auckland, Faculty of Medical and Health Sciences, 85 Park Road, Grafton, Auckland 1023, New Zealand; ^3^Waitemata Cardiology, 181 Shakespeare Road, Milford, Auckland 0620, New Zealand

## Abstract

We present a unique case study report of a male individual with a history of mild nonischaemic cardiomyopathy, with no ventricular ectopy, that at the age of 76 years sustained multiple concussions (i.e., mild traumatic brain injury) within a week of each other. Concussion symptoms included cognitive difficulties, “not feeling well,” lethargy, fatigue, and signs of depression. He was later medically diagnosed with postconcussion syndrome. The patient, WJT, was referred for cardiac and neurological assessment. Structural neuroimaging of the brain (MRI) was unremarkable, but electrocardiography (ECG) assessments using a 24-hour Holter monitor revealed significant incidence of ventricular ectopy (9.4%; 9,350/99,836 beats) over a period of 5–6 months after injury and then a further increase in ventricular ectopy to 18% (15,968/88,189 beats) during the subsequent 3 months. The patient was then prescribed Amiodarone 200 mg, and his ventricular ectopy and concussion symptoms completely resolved simultaneously within days. To the authors' knowledge, our study is the first to show a direct link between observable and documented cardiac dysregulation and concussion symptomology. Our study has important implications for both cardiac patients and the patients that sustain a concussion, and if medically managed with appropriate pharmacological intervention, it can reverse ventricular ectopy and concussion symptomology. More research is warranted to investigate the mechanisms for this dramatic and remarkable change in cardiac and cerebral functions and to further explore the brain-heart interaction and the intricate autonomic interaction that exists between the extrinsic and intracardiac nervous systems.

## 1. Introduction

There is a significant body of literature to show that neuroautonomic cardiovascular dysregulation can occur as a result of a concussion or mild traumatic brain injury (mTBI). Much of this research has used heart rate variability, blood pressure variability, cerebral blood flow metrics, and baroreflex sensitivity to try to diagnose the extent of autonomic nervous system (ANS) dysregulation [1–12]. Mechanisms such as dynamic cerebral autoregulation, neurovascular coupling, and cerebrovascular reactivity to carbon dioxide have been proposed to account for cognitive disturbances and symptoms associated with mTBI [4, 13–15].

Furthermore, there is an accumulation of research that has investigated the brain-heart (heart-brain) relationship and interaction, specifically an advancing field of study called “neurocardiology” and the neurocardiac axis theory [16–21]. Neurocardiology explores the (patho) physiological interaction between the brain and the cardiovascular system [17, 20, 22, 23] and how the ANS function is involved [18, 24, 25]. We propose here that this brain-heart interaction could explain the apparent randomness of sudden cardiac events experienced in our case study patient.

The stuctural and functional organization of cardiac innervation in the neurocardiac axis is illustrated by Shivkumar et al [19]. Their illustration provides a schematic representation for the increasing evidence that the intracardiac nervous system and afferent feedback from the heart to higher brain centres may affect efferent output back to the heart and modulate cardiac tissue electrophysiology [19, 21]. Chemosensory feedback and mechanosensory feedback to higher brain centres also involve afferent neurons throughout the nodose and dorsal root ganglia and provide an important link from the heart to higher brain centres suggesting that there is a “brain within the heart” [18].

Thus, the purpose of this case study was to report two novel findings as follows: (1) mTBI caused cardiac arrhythmias, which is consistent with the theory of a neurocardiac axis and (2) pharmacological intervention with Amiodarone administration completely reversed cardiac dysregulation and concussion symptomology simultaneously within days.

## 2. Case History Presentation

The earliest medical files on patient WJT on record is dated 4^th^ December 1997 (age 57 yrs) to show that he had minor symptoms of ventricular ectopy, left bundle branch block (LBBB), and mild nonischaemic cardiomyopathy. When assessed almost 8 years later (6^th^ July 2005), his cardiovascular examination was not significantly changed and was normal with no carotid bruits, normal jugular venous pressure, normal heart sounds with no murmurs, and clear lung fields. He was not taking any medications (Sotalol was taken very rarely as he was significantly symptomatic with this medication, so he avoided it). His ECG demonstrated a normal sinus rhythm with a LBBB. No ectopy was seen. However, a dipyridamole sestamibi myocardial nuclear perfusion scan was ordered because the patient experienced an increased frequency of atypical chest discomfort during the previous couple of months. The scan was abnormal, documenting an ejection fraction (EF) of 39% with a dilated left ventricle and a left ventricular end-diastolic volume (LVEDV) of 171 mL. There was a mild reduction in tracer uptake in the septum, which was consistent with the LBBB artefact. No definite ischaemia was identified. The patient was started on Inhibace 0.5 mg. The goal was ultimately to increase his angiotensin-converting enzyme (ACE) inhibitor to a maximal dose and reconsider the role of a *β*-blocker [26].

His follow-up assessment in October 2005 included an angiogram that showed minimal coronary disease and surprisingly normal LV function with an EF of 73% and normal left ventricular volume when assessed using LV ventriculography [27, 28]. The small dose of Inhibace was really insufficient to have caused such a substantial improvement. It was postulated that WJT had a subclinical episode of myocarditis, resulting in his increased symptoms and reduced systolic function. This appears to have resolved over this short-term period. WJT maintained his Cartia Aspirin 100 mg and increased his Inhibace to 2.5 mg daily as his blood pressure was 144/85 mmHg on the day of assessment.

Regular follow-ups continued every 6–12 months from 2005 to 2013 that included ECG, echocardiography, chest examinations, and prescription drug management as per normal medical practice in an ageing patient with some history of cardiovascular disease. During this period, WJT was generally well and unlimited in terms of his activities of daily living, including hiking, skiing, and hunting. He was maintained on various medications to ensure an active healthy lifestyle and to control for his history of mild nonischaemic cardiomyopathy. Medications over this period of time included Inhibace 2.5 mg, short-acting Diltiazem 30 mg, Metoprolol 50 mg, angiotensin receptor blocker, Aspirin, Atacand (Candesartan) 8 mg, and Diazepam 2 mg, and his Candesartan 8 mg was switched at that stage to Losartan 25 mg. Cardiac structure and function remained typically the same with normal systolic and diastolic function, interatrial septum intact, EF%, and no valvular disease (left ventricular end-diastolic diameter (LVEDD) = 5.8 cm, end-systolic diameter (ESD) = 3.9 cm, interventricular septum (IVS) = 1.1 cm, left ventricular posterior wall thickness (LVPW) = 0.9 cm; left atrium measures 20 cm^2^; EF 70%). Blood pressure (varied from 138/85 to 145/95 mmHg) and cholesterol profile (total 4.7 to 5.0 mmol/L, HDL 1.2 to 1.5 mmol/L, and LDL 2.8 to 2.9 mmol/L) remained relatively healthy (now age 73 yrs).

**Figure 1:**
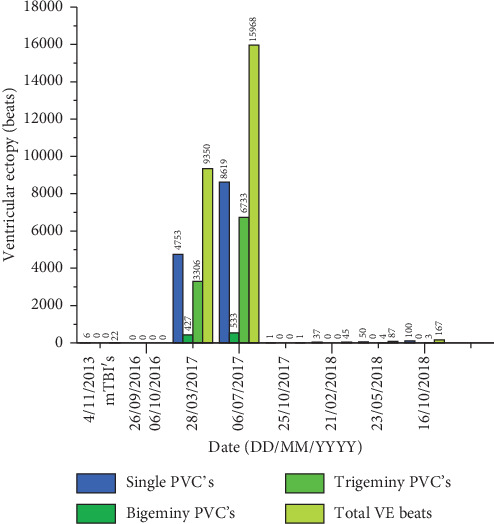
Total number of single premature ventricular contractions (PVC), bigeminy, trigeminy, and total ventricular ectopic (VE) beats assessed during the 24-hour Holter monitor from April 2013 to October 2018. The two concussions (mTBI) are dated in the Figure.

In November 2013, patient WJT had a 24-hour Holter that revealed only a very minimal amount of ectopy (Table 1, Figure 1) although this was indeed symptomatic and he was quite sensitive to perceiving the ectopic beats. His exercise stress echocardiogram confirmed findings consistent with his known longstanding mild cardiomyopathy, but he achieved a good workload (8 minutes of the Bruce protocol without symptoms limiting the exercise).

### 2.1. Concussion Incident: 26^th^ September and 6^th^ October 2016

The patient WJT was 76 years old when he sustained two concussions within approximately 10 days: the first on 26^th^ September and the second on 6^th^ October 2016. These were later medically diagnosed as a concussion or mTBI. Because of his symptoms of increased ectopy and change in perception of cognitive function, WJT contacted us with concerns about his increased ectopy and malaise. WJT reported that these symptoms had been progressing over the past 5-6 months since his concussions. Symptoms included cognitive “deterioration,” “not feeling well,” headache, “lack of energy and fatigue,” “irritability,” signs of depression, and “acute audio sensitivity and poor visual acuity” which were the two most extreme, persistent and troubling symptoms that WJT experienced.

On 28^th^ March 2017, he undertook a 24-hour Holter monitor which showed a high burden of ventricular ectopy at 9.4% (9,350/99,836 beats). This is in stark contrast to his 24-hour Holter in 2013 where there were negligible ectopic beats (Table 1; Figure 1). The patient was started on a *β*-blocker Metoprolol daily 47.5 mg, but he was advised to stop taking angiotensin receptor blocker Losartan as his blood pressure would not sustain taking both medications [29].

In the coming months (May 2017), he performed another resting echocardiogram and exercise stress echocardiogram. Resting echocardiogram revealed that the left ventricle was normal in size and had low-normal systolic function with an estimated EF of 50%. There was considerable beat-to-beat variability depending on the degree of ectopy. Measurements included LVEDD = 5.1 cm; ESD = 3.1 cm; IVS = 1.1 cm; LVPW = 1.1 cm; and EF 50%. The patient completed 8 minutes of the Bruce protocol achieving 95% maximal predicted heart rate with blood pressure rising from rest at 138/70 to 182/85 mmHg at the termination of exercise. Again, adverse cardiac symptoms did not limit this test. WJT felt well throughout the stress test, and the resting monomorphic ventricular ectopy was resolved at peak stress. The ECG showed LBBB throughout the assessment. The impression of the stress echocardiogram at peak exercise suggested that there was an acceptable augmentation of wall thickening in all myocardial segments without clear evidence for ischaemia. Image quality was good. Thus, the exercise stress echocardiograph demonstrated a mild nonischaemic cardiomyopathy as previously known without significant ischaemia at stress and no significant change from 2013.

Although his May–June 2017 echocardiographic assessments and follow-up showed only minor changes from November 2013, the high percentage of ventricular ectopy (9.4%) was problematic for the patient. He stated that he had periods of depression. He did not feel right and expressed concerns about his cognition; again, this was likely a result of his two back-to-back concussions. Based on his description of symptoms that he was experiencing, it is likely that he had been suffering from postconcussion syndrome [30, 31]. The goal at this point was to try to suppress the significant number of ectopic beats. During the ensuing time period, WJT continue with the Metoprolol 47.5 mg daily and was referred for another 24-hour Holter monitoring.

The patients 24-hour Holter (6^th^ July 2017) showed another significant increase in ventricular ectopy at 18.1% (Table 1; Figure 1). This was a significant increase from 9.4% recorded only 3 months prior on 28^th^ March 2017. This increased ventricular ectopy also matched his persistent symptoms associated with postconcussion syndrome. Furthermore, there was a significant increase in both bigeminy and trigeminy ventricular ectopic beats (Figure 1) that was not apparent before his concussion head injury. To manage the significant increase in ventricular ectopy, the patient was prescribed Amiodarone 200 mg dose to suppress the high rate of ventricular ectopy that we believed was symptomatic and likely to be aggravating the preexisting modest LV dysfunction and his postconcussion symptoms. Patient WJT remained on Amiodarone from July 2017 to October 2017.

Within days of Amiodarone administration, our patient began feeling better, and interestingly, and remarkably, the patient reported that his intermittent cognitive impairment and mTBI/postconcussion symptomology that he experienced after his head injury had completely resolved. However, what patient WJT found to be most remarkable was that within days, “my most troubling concussion symptoms totally disappeared and simultaneously I could no longer physically detect any pronounced ventricular ectopic beats,” which he was very sensitive to.

His follow-up 24-hour Holter monitor (25^th^ October 2017) confirmed an absence of ventricular ectopy (0%; 1/89,189 beats). The resolution of the ventricular ectopy was successfully managed with Amiodarone, as was his postconcussion symptoms. He remained stabilised on the 200 mg dose of Amiodarone.

Because of the concern regarding the long-term side effects of Amiodarone, his dosage was dropped 100 mg and kept for 3 months and then dropped again to 50 mg thereafter (February 2018). WJT felt completely well on the 50 mg dose of Amiodarone for a good period of time (March to June 2018). Despite the substantial dose reduction, he remained free of any important ventricular ectopy with a very low burden of ventricular dysrhythmia (0.1%; 87/100, 487 beats) on his most recent 24-hour Holter monitor (23^rd^ May 2018). At this time, the patient was stopped from taking Amiodarone completely and prescribed his longstanding Losartan 25 mg. His follow-up 24h-hour Holter monitor on 16^th^ October 2018 showed that ventricular ectopy was 0.2% (167/98,937 beats). At this time, no further postconcussion symptoms were reported.

### 2.2. Study Design and Methods

This was a retrospective study that was approved by a University Research Ethics Board (REB #2018-113), and all work was conducted in accordance with the Declaration of Helsinki (1964). The patient WJT provided his written informed consent to have all of his medical records available for this case study report. Patient WJT DOB is 23^rd^ September 1940, and he was 76 years of age at the time of his two concussions within 10 days of each (26^th^ September and 6^th^ October 2016).

The data were acquired from all medical records from 1997 to 2018 and put into Microsoft Excel spreadsheet (Microsoft Office, Microsoft Corporation, Seattle, USA) for ease of data management and interpretation. The ventricular and supraventricular (atrial) data were taken from the 24-hour Holter monitor (Phillips Holter Monitoring Recorder, Phillips, New Zealand). Heart rate variability (HRV) data were taken from the printed Holter monitor report. We attempted to get the raw ECG data to conduct our own HRV analysis, but this was not possible due to restrictions of the manufacturer. HRV was not conducted during the preconcussion 24-hour Holter monitoring (4^th^ November 2013). However, the HRV software was consistent for all recording dates after the concussions. Fast Fourier transformation using Welch's periodgram method for frequency-domain and time-domain spectral analyses was used to determine HRV metrics [32].

### 2.3. Results

**Figure 2:**
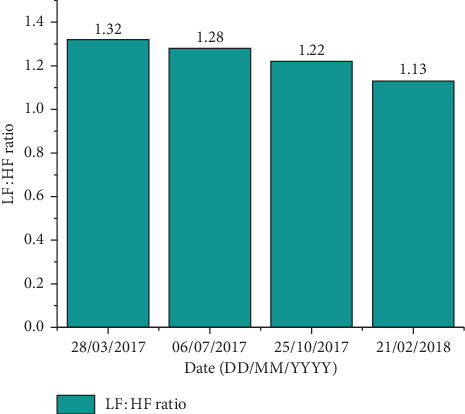
Ratio of low frequency (LF) to high frequency (HF) power (LF : HF) heart rate variability after the patient sustained his two mTBI's (September and October 2016).

The results for this case study report are presented in Tables 1 and 2 and Figures 1 and 2. As noted in Table 1 and Figure 1, there was a significant increase in ventricular (VE) and supraventricular (SVE) ectopy within months of the patient sustaining his two concussions in September and October 2016. In addition to the VE and SVE, single prematureatrial contractions (PACs) and bigeminy and trigeminy beats were also significantly increased following the concussion incidents.  Table 2 and Figure 2 represent the heart rate variability (HRV) data. HRV was observed through spectral power analysis frequencies, being ultra-low frequency (0–0.003 Hz), very low frequency (VLF; 0.003–0.04 Hz), low frequency (LF; 0.04–0.15 Hz), and high frequency (HF; 0.15–0.4 Hz) [33]. Results showed that the LF : HF ratio, as assessed by the power of LF to HF, decreased with the reduction of ventricular ectopic beats.

The nonlinear analysis of the ECG has been used to derive HRV [34], by analyzing the variation in the R to R peaks of the ECG waveform. HRV has been used to reflect sympathetic and parasympathetic control and neurocardiac autonomic function [35, 36]. Changes in HRV are due to alterations in both low (LF) and high (HF) frequency [35, 37], and research has shown that there is an increase in the LF : HF ratio in individuals with persistent ventricular ectopic beats [38]. For our patient, ventricular ectopic events were highest on 28^th^ March 2017 (9.4% ventricular ectopy) and 6^th^ July 2017 (18% ventricular ectopy). Furthermore, this increase in the LF : HF ratio with an increase in ventricular ectopy does suggest a potential impairment in the sympathovagal balance of HRV [39]. However, over the following one-year period, the LF : HF ratio decreased from 1.32 to 1.13 (Figure 2), suggesting a decreased reliance on LF dominance, and improved HRV/ANS function. LF power is a net effect of several intrinsic modulatory factors from both sympathetic and parasympathetic branches of the ANS, vagally mediated baroreflex, and even some respiratory influences at lower respiratory rates [8, 40]. All other time-domain analysis variables were unchanged from 28^th^ March 2017 to 21^st^ February 2018.

## 3. Discussion

This is the first study, to our knowledge, to show direct evidence that acute mTBI and subsequent postconcussion syndrome symptomology can cause cardiac arrhythmias and that the symptoms of mTBI and ventricular dysregulation were completely abolished within days with pharmacological intervention of Amiodarone in our patient. This is a novel finding.

It has been proposed in the brain-heart link, that there are intracardiac sensory neurons that relay sensory information back to the brain via the dorsal root and nodose ganglion [18, 19, 41]. These sensory signals then communicate between the medulla and the higher brain centres. Therefore, our results provide further support that there is afferent feedback from the heart to the brain and then efferent outflow to the heart to accommodate normal cardiac function [18, 19, 42]. The complete resolution of postconcussion symptoms (within days of Amiodarone administration) in patient WJT supports this notion.

The precise mechanism(s) for the surprisingly high increased ventricular ectopy (18.1%) in patient WJT stemming from his mild head injury is not completely known, but it has been proposed that higher brain centres or brain stem regions may participate in the arrhythmogenesis [20, 21]. As stated previously, mTBI can cause ANS dysregulation, and thus, both sympathetic and parasympathetic innervations to the heart are compromised [8, 9, 43]. Research by Shivkumar et al. [19] would support this contention, based on their schematic illustration showing that the intrinsic cardiac nervous system has its own sympathetic and parasympathetic systems, thus suggesting that the electrophysiological system responsible for coordinated normal sinus rhythm was no longer intact in our patient following his mTBI. Research by Taggart et al. [20, 21] suggests that autonomic nerve activity may influence electrophysiology through myocardial ion channels, pumps and transporters, and intracellular signalling processes. Furthermore, recent research has shown that PVCs can alter the activity of intrinsic cardiac ganglion neurons in vivo [42, 44], which could have added to the ventricular ectopy experienced by our patient WJT. Hamon et al. [42] used a porcine model and cardioneural mapping to demonstrate that PVCs are powerful modulators of vagal afferent activity and significantly increase sensory neural input to the brain stem. They also demonstrated that PVCs can activate both chemosensory and mechanosensory neurons in the inferior vagal ganglia. Finally, their research showed that nodose ganglion sensory neurons are adaptable, can display memory in response to a cardiac stimulus, and can prolong cardiac dysregulation caused by the arrhythmias. This is likely caused by the release of reactive oxygen species that can activate chemosensitive neurons by altering Ca^+2^ channels and mitochondrial function [45–47]. This would potentially explain why ventricular ectopy increased 9.4% (March 2017) after the initial concussions and then continued to increase dramatically to 18.1% over the next 3-4 months (July 2017). Thus, our data would support the contention promoted by Hamon et al. [42] that processing and integration of sensory information are occurring at different levels within the ANS, including the intrinsic cardiac nervous system (ICNS). This would offer an elegant mechanism to ensure fine-tuned regulation of efferent neural signals from the brain to the heart.

The precise physiological mechanism(s) for the remarkable reversal in ventricular ectopy and mTBI symptomology in our patient is not completely elucidated from our study data, but our results showed that pharmacological intervention with Amiodarone reestablished the cardiac intracellular milieu and autonomic function. Amiodarone is a class III antiarrhythmic agent, is a multichannel blocker that has been shown to block potassium rectifier currents responsible for cardiac repolarization [48], and has the ability to act on Ca^+2^ and sodium channels and act as a *α*- and *β*-blocker [49–52]. Thus, reestablishment of the chemical and ionic homeostasis in the heart, which likely has a positive effect on the intrinsic cardiac nervous system, communicates back to the higher brain centres via the nodose and dorsal root ganglion and influences the ANS function. It has been suggested that the autonomic nerve activity can influence cardiac electrophysiology through intracellular signalling processes [21].

In addition to working directly on cardiac tissue to reverse the ventricular arrhythmia, Amiodarone has been shown to alter cerebral chemical channels too [53]. Using a stroke mouse model, Kotoda et al. [53] showed that the administration of Amiodarone prior to initiating a stroke was neuroprotective. Therefore, it is conceivable that Amiodarone had a positive effect on both the brain and heart receptors in helping to reestablish cellular homeostasis of our patient with postconcussion symptomology. Further research in this specific area is warranted.

### 3.1. Heat Rate Variability (HRV)

In this study, we also used HRV as a surrogate measure to assess autonomic nervous system function [8, 43, 44, 54–56]. When we assessed the ECG data for HRV, the results showed that the LF : HF ratio (Figure 2) was elevated when patient WJT was experiencing his postconcussion symptoms and increased ventricular ectopy (March–July 2017). This increased LF : HF ratio suggests that there was an impairment of the sympathovagal balance [39, 57]. The dysregulation of the ANS has been documented previously in sport-related concussion [7, 15, 43], and this is what likely contributed to his postconcussion symptoms [58]. Because of the tight relationship between the brain and the heart [18, 19], this ANS dysregulation likely contributed to the increased ventricular ectopy. It is thus conceivable that the intracardiac nervous system was significantly altered [19] and added to the observed cardiac impairment, as stated above [42, 44]. Collectively, this further implies that PVCs are powerful modulators of cardiac vagal afferent neurotransmission and can have an effect on the HRV response. Our observations would support recent research by Askin et al. [59] using the 24-hour Holter data that showed patients with elevated blood pressure and PVCs had an increased LF : HF ratio. Salavatian et al. [44] used a porcine model to show that parasympathetic withdrawal (HF decrease) was associated with PVCs. Taken together, these research studies suggest that the sympathetic nervous system is dominant under conditions of PVCs.

Concomitant with the administration of Amiodarone for our patient WJT, these results showed that with the remarkable reduction of ventricular ectopy, there was also a concomitant reduction of LF : HF ratio (Figure 2). This indicates an improved ANS function and would help to explain the disappearance of cognitive symptoms that plagued patient WJT for 6–10 months after injury (Amiodarone was administered in July 2017).

In conclusion, this case study research suggests that concussion or mTBI can cause physiologic manifestations to cardiac tissue directly and support the literature showing a direct link between the heart and the brain during pathology. The mechanism for the reported cardiac dysregulation in our patient was likely related to ANS dysfunction. Our novel finding is that when we treated the heart with Amiodarone, all concussion symptoms and ventricular ectopy disappeared completely within days, further documenting the intricate relationship between the brain and the heart. Finally, the neurocardiac axis theory and neurogenic stunned myocardium phenomenon, both described above in the Introduction, could partly explain the brain-heart link and interaction and can thus pave the way to a better understanding and management of mTBI [17].

## Figures and Tables

**Table 1:** Ventricular and supraventricular ectopy data from the 24-hour Holter monitor from April 2013 to October 2018.

Holter session date (day/month/year)			4/11/2013	mTBI 09/2016 10/2016	28/03/2017	06/07/2017	25/10/2017	21/02/2018	23/05/2018	16/10/2018
Ventricular ectopy	Ventricular runs	Total VE beats	22		9,350	15,968	1	45	87	167
		% VE	0.00%		9.40%	18.10%	0.00%	0.00%	0.10%	0.20%
		#No. of VE runs	0		0	0	0	0	0	0
		VE beats	0		0	0	0	0	0	0
		VE longest beat	0		0	0	0	0	0	0
		VE fastest beat	0		0	0	0	0	0	0
		Triplets	0		1	0	0	0	0	1
		Couplets	3		6	4	0	0	2	5
		Single PVCs	6		4,753	8,619	1	37	50	100
		Interpreted PVCs	0		1	2	0	0	7	5
		R on T PVCs	0		0	0	0	0	0	0
		Single VEs	10		843	69	0	8	21	46
		Late VEs	0		5	4	0	0	1	0
		Bigeminy	0		427	533	0	0	0	0
		Trigeminy	0		3,306	6,733	0	0	4	3

Supraventricular ectopy	Atrial runs	Total SVE beats	66		99	63	34	107	175	119
		% SVE	0.10%		0.10%	0.10%	0.00%	0.10%	0.20%	0.10%
		No. of atrial runs	2		2	0	0	3	2	1
		AR beats	15		13	0	0	11	12	4
		AR longest	12		10	0	0	5	7	4
		AR fastest	107		128	0	0	112	119	132
		Atrial pairs	1		2	2	1	5	1	5
		Drop	0		0	0	0	0	0	0
		Late	1		3	3	0	1	1	1
		Longest N–N (s)	1.6		1.5	1.7	1.5	1.7	1.4	1.5
		Single PACs	48		79	56	28	82	160	101
		Bigeminy	0		0	0	4	3	0	3
		Trigeminy	0		0	0	0	0	0	0

The two concussions (mTBI) are dated in the table. VE = ventricular ectopy; PACs = premature atrial contractions; PVCs = premature ventricular contractions; R on T (PVC) phenomenon = superimposition of an ectopic beat on the T-wave of a preceding beat; VE = supraventricular ectopy; AR = atrial runs. Amiodarone was administered following the Holter monitoring on 06/07/2017.

**Table 2:** Heart rate and heart rate variability (HRV) data from the 24-hour Holter monitor from April 2013 to October 2018.

HRV metrics	4/11/2013	mTBI 09/2016 10/2016	28/03/2017	06/07/2017	25/10/2017	21/02/2018
Total number beats	101,126		99,836	88,189	89,189	94,663
Minimum HR (bpm)	43		45	44	44	42
Average HR (bpm)	70		70	61	64	66
Maximum HR (bpm)	128		114	94	117	109
ASDNN 5 (ms)	0		45.1	44.2	43.2	45.6
SDANN 5 (ms)	0		169.7	132.3	195.4	182.4
SDNN (ms)	0		177.3	149.4	199.4	183.5
Ultra low (0–0.003 Hz) (ms^2^)	0		3,880	3,390	3,938	3,984
Very low (0.003–0.04 Hz) (ms^2^)	0		9,135	7,221	7,446	8,995
Low (0.04–0.15 Hz) (ms^2^)	0		6,458	5,906	6,203	6,207
High (0.15–0.4 Hz) (ms^2^)	0		4,895	4,626	5,100	5,491
Total (0–0.5 Hz) (ms^2^)	0		25,745	22,297	24,034	26,188
Ultra low (0–0.003 Hz) (%)	0		15.1%	15.2%	16.4%	15.2%
Very low (0.003–0.04 Hz) (%)	0		35.5%	32.4%	31.0%	34.3%
Low (0.04–0.15 Hz) (%)	0		25.1%	26.5%	25.8%	23.7%
High (0.15–0.4 Hz) (%)	0		19.0%	20.7%	21.2%	21.0%
LH : HF	—		1.32	1.28	1.22	1.13

The two concussions (mTBI) are dated in the table. No HRV data were available on 4-11-2013. ASDNN = average of the standard deviations of all R-R intervals for all 5 min segments in the 24 h recordings; SDANN = standard deviation of all averaged normal sinus R-R intervals for all 5 min segments in the 24 h recordings; SDNN = standard deviation of all normal sinus R-R intervals in the entire 24 h recording. Ultra-low frequency (ULF), 0–0.003 Hz; very low frequency (VLF), 0.003–0.04 Hz; low frequency (LF), 0.04–0.15 Hz; high frequency (HF), 0.15–0.4 Hz; and total frequency (TF), 0–0.5 Hz.
